# Immortalized Alzheimer’s Disease Astrocytes: Characterization of Their Proteolytic Systems

**DOI:** 10.1007/s12035-023-03231-z

**Published:** 2023-02-02

**Authors:** Chunmei Gong, Laura Bonfili, Yadong Zheng, Valentina Cecarini, Massimiliano Cuccioloni, Mauro Angeletti, Giulia Dematteis, Laura Tapella, Armando A. Genazzani, Dmitry Lim, Anna Maria Eleuteri

**Affiliations:** 1 School of Biosciences and Veterinary Medicine, University of Camerino, Via Gentile III da Varano, 62032 Camerino, MC Italy; 2 Department of Pharmaceutical Sciences, Università del Piemonte Orientale, Via Bovio 6, 28100 Novara, Italy

**Keywords:** Alzheimer’s disease, Astrocytes, Ubiquitin–proteasome system, Autophagy, 4-Phenylbutyric acid

## Abstract

Alzheimer’s disease (AD) is a progressive neurodegeneration with dysfunctions in both the ubiquitin–proteasome system (UPS) and autophagy. Astroglia participation in AD is an attractive topic of research, but molecular patterns are partially defined and available in vitro models have technical limitations. Immortalized astrocytes from the hippocampus of 3xTg-AD and wild-type mice (3Tg-iAstro and WT-iAstro, respectively) have been obtained as an attempt to overcome primary cell line limitations and this study aims at characterizing their proteolytic systems, focusing on UPS and autophagy. Both 26S and 20S proteasomal activities were downregulated in 3Tg-iAstro, in which a shift in catalytic subunits from constitutive 20S proteasome to immunoproteasome occurred, with consequences on immune functions. In fact, immunoproteasome is the specific complex in charge of clearing damaged proteins under inflammatory conditions. Parallelly, augmented expression and activity of the lysosomal cathepsin B, enhanced levels of lysosomal-associated membrane protein 1, beclin1, and LC3-II, together with an increased uptake of monodansylcadaverine in autophagic vacuoles, suggested autophagy activation in 3Tg-iAstro. The two proteolytic pathways were linked by p62 that accumulated in 3Tg-iAstro due to both increased synthesis and decreased degradation in the UPS defective astrocytes. Treatment with 4-phenylbutyric acid, a neuroprotective small chemical chaperone, partially restored proteasome and autophagy-mediated proteolysis in 3Tg-iAstro. Our data shed light on the impaired proteostasis in 3Tg-iAstro with proteasome inhibition and autophagic compensatory activation, providing additional validation of this AD in vitro model, and propose a new mechanism of action of 4-phenylbutyric acid in neurodegenerative disorders.

## Introduction

Alzheimer disease (AD) is the most common type of dementia that has become a rapidly increasing public health concern. The biological construct that helps defining AD comprises the deposition of amyloid-β (Aβ) plaques, pathological tau phosphorylation, and neurodegeneration [1, 2]. A growing body of evidence identified that oxidative stress, chronic inflammation, mitochondrial dysfunction, and endoplasmic reticulum (ER) stress have a role in AD development [3, 4].

Astrocytes, the most abundant glial cells in the central nervous system (CNS), are involved in numerous aspects of CNS physiology. Specifically, astrocytes act as scavengers for reactive oxygen species and supply cysteine precursor for neuronal glutathione [5]. Astrocytes are involved in the removal of toxins, production and release of trophic factors, regulation of neurotransmitters, and ion concentrations, thereby maintaining the overall cell homeostasis, including optimal synaptic glutamate levels and neuroimmune status [6, 7]. During AD progression, astrocytes undergo complex alterations becoming first asthenic and hypotrophic, while later they turn to be reactive and hypertrophic, mostly around developing senile plaques [7, 8]. During pathological remodeling, astrocytes lose their homeostatic and neuroprotective functions, thus representing a potential preventative or therapeutic target in the preclinical phase of AD.

Cells possess two major intracellular proteolytic pathways, namely the ubiquitin–proteasome system (UPS) and autophagy. The UPS is the major degradation system used by cells and involved in the disposal of misfolded and unfolded proteins accumulated in the endoplasmic reticulum (ER) [9]. The eukaryotic 26S proteasome is a large, multi-catalytic protease complex in charge of the removal of intracellular misfolded, oxidized, or aggregated ubiquitin-tagged-proteins, in an ATP-dependent manner [10]. The catalytic core of this structure is the 20S proteasome, consisting of two rings made of seven α subunits, flanking two superimposed rings made of seven β subunits. Among β-subunits, only three (β1, β2, β5) possess an intact active site and cleave proteins into the proteolytic chamber with a total of six active sites inside a functional proteasome [11–13]. Specifically, subunit β1 is associated with the caspase-like activity or peptidylglutamyl-peptide hydrolysing (PGPH) activity; subunit β2 exerts a trypsin-like (T-L) activity; subunit β5 is related to the chymotrypsin-like (ChT-L) activity, but given its tendency to cleave after small neutral and branched side chains also the small neutral amino acid preferring (SNAAP) and branched-chain amino acid preferring (BrAAP) activities can be assigned to this subunit [14]. Numerous data have demonstrated that AD and other neurodegenerations are characterized by an impaired UPS functionality and that amyloid aggregates are able to further inhibit such complex [15]. However, in cells exposed to IFN-γ or tumor necrosis factor-α (TNF-α), the 20S proteasome is converted into the immunoproteasome with its constitutive catalytic subunits, β1, β2, and β5, being replaced by the inducible subunits β1i, β2i, and β5i [16]. In immunoproteasome, β2i and β5i express trypsin-like and chymotrypsin-like enzymatic activity similarly to the constitutive counterparts. β1i can exert chymotrypsin-like activity, differently from the constitutive β1 which is associated to PGPH activity [17]. Due to distinct cleavage sites, inducible β subunits can hydrolyze proteins in a distinct manner with respect to constitutive subunits and can generate peptides binding major histocompatibility complex (MHC) class I molecules, playing a role in antigen presentation process [18]. Depending on the tissue and cell type, different proteasome arrangements can coexist [19]. Previous works have shown that immunoproteasome expression is related not only to neuroinflammation in AD but also to aging [20].

Autophagy is a highly conserved lysosome-dependent proteolytic system that attempts to restore cellular homeostasis through the degradation of unfolded/misfolded or aggregated proteins and damaged subcellular organelles [21]. The autophagy process involves several steps, including nucleation of the isolation membrane named phagophore, expansion and closure of the phagophore, fusion between the resulting autophagosomes and multivesicular endosomes or lysosomes, and degradation of autophagosome contents [22]. Autophagy is involved in AD pathogenesis [23]. Specifically, it is involved in Aβ aggregates clearance and it preserves neuronal function [24]. Cathepsins B and L are cysteine proteases contained in lysosomes with a role in AD pathogenesis, being involved in cholesterol metabolism, Aβ peptide degradation, and amyloid precursor protein (APP) processing, thus representing a therapeutic target in neurodegenerations [25]. Changes in cathepsins activity are normally found in aging neurons and are considered as a cause of age-related neuropathologic variations. Increased cathepsin B levels have been previously observed in AD animal models. Cathepsin B is associated with amyloid plaques in AD brains and has been suggested to be responsible for the increased Aβ production. Conversely, cathepsin L activity increases α-secretase-mediated non-amyloidogenic pathway [25, 26]. Lysosomal-associated membrane protein 1 (LAMP1) is considered a lysosome marker, whereas microtubule-associated protein light chain 3 (LC3) is an indicator of autophagosome formation, which preferentially interacts with the autophagy-adaptor protein SQSTM1/p62, thus mediating selective degradation during autophagy [27]. p62 has been demonstrated to work as a receptor for ubiquitinated proteins and organelles to be degraded by lysosomal enzymes. It is a common component of protein inclusions in several neurodegenerative disorders, among them neurofibrillary tangles in AD [28]. Moreover, beclin-1 plays an important role in membrane isolation and nucleation, contributing to the formation of early autophagosomes [29]. Interestingly, deletion of BECN1 in AD animal models enhanced the intracellular and extracellular Aβ loads [30]. However, molecular mechanisms underlying defective proteolysis in astrocytes in AD are not well clarified.

Immortalized astrocytic cell lines were generated from hippocampi of 3xTg-AD mice, a well-established AD mouse model, and from the wild-type counterpart [31]. These lines named 3Tg-iAstro and WT-iAstro faithfully reproduce the features of primary astrocytic cultures from 3xTg-AD mice and WT mice in terms of gene profiling, proteostasis, Ca^2+^ signaling, and ER-mitochondria interaction [31–33], but further analysis must be carried out to reach a complete characterization of such cell lines.

Interestingly, chaperones have been shown to reduce levels of misfolded proteins, thus minimizing the accumulation of aggregates and their downstream pathological consequences [34]. The chemical chaperone, sodium 4‐phenylbutyric acid (4‐PBA), is a small‐molecular‐weight and blood‐brain barrier permeable fatty acid, able to regulate ER stress, attenuate cell damage, and help unfolded protein remodeling [34–36]. It has been already used for the treatment of urea cycle disorders and has been recently considered with high potential as a new drug for preventing cognitive decline [37].

In the current study, the proteolytic pathways of both 3Tg-iAstro and WT-iAstro have been characterized, focusing on proteasome subunit composition and functionality and on key autophagic markers [38]. In addition, the ability of 4‐PBA treatment to affect the interconnected proteolytic systems in both WT-iAstro and 3Tg-iAstro has been investigated, as a possible mechanism of action of the neuroprotective compound.

## Material and Methods

### Reagents and Chemicals

The substrates Suc-Leu-Leu-Val-Tyr-7-Amino-4-methylcoumarin (AMC), Z-Leu-Ser-Thr-Arg-AMC, Z-Leu-Leu-Glu-AMC for assaying the chymotrypsin-like (ChT-L), trypsin-like (T-L), and peptidyl glutamyl-peptide hydrolyzing (PGPH) proteasomal activities were purchased from Sigma-Aldrich S.r.L. (Milano, Italy). Z-Gly-Pro-Ala-Leu-Ala-MCA to measure BrAAP activity was from Biomatik (Cambridge, Ontario). Aminopeptidase N (EC 3.4.11.2) was purified from pig kidney [39]. Cathepsin B and cathepsin L substrates (Z-Arg-Arg-AMC and Z-Phe-Arg-7-amino-4-trifluoromethyl-coumarin (AFC) respectively), cathepsins inhibitors (CA074Me and N-(1-Naphthalenylsulfonyl)-Ile-Trp-aldehyde), and monodansylcadaverine (MDC) were obtained from Sigma-Aldrich S.r.L. (Milano, Italy). Media and chemicals used for cell cultures were purchased from Enzo Life Sciences, Inc. The membranes for western blot analyses were purchased from Millipore (Milan, Italy). Proteins immobilized on polyvinylidene fluoride membranes were detected with the enhanced chemiluminescence (ECL) technique (Amersham Pharmacia Biotech, Milan, Italy) on a ChemiDoc MP, Chemiluminescence system (Biorad, Italy).

### Cell Lines

Generation of immortalized astrocytes from hippocampi of WT and 3xTg-AD mice (WT-iAstro and 3Tg-iAstro cells) was described elsewhere [31]. Briefly, the immortalized cell lines were generated from separate primary astrocyte cell cultures deriving from both WT and AD mice. For immortalization, primary astroglia cultures were depleted of microglial cells by magnetic-assisted cell sorting using anti-CD11b-conjugated microbeads obtaining a population of highly purified astrocytes. Cells were transduced using retrovirus expressing Simian Virus 40 large T antigen (SVLT). Transformed cells were selected in G418, amplified, and stabilized for 12 passages prior to characterization. No clonal selection was performed to maintain the natural heterogeneity of the cultures.

In this study, three independent WT-iAstro and three independent 3Tg-iAstro lines were grown in complete culture media containing Dulbecco’s modified Eagle’s medium (DMEM; Sigma-Aldrich, Cat. No. D5671) supplemented with 10% fetal bovine serum (Gibco, Cat. No. 10270), 2 mM L-glutamine (Sigma-Aldrich), and 1% penicillin/streptomycin solution (Sigma-Aldrich). Cells were incubated with growth medium at 37 °C equilibrated with 95% air and 5% CO_2_ in flasks or dishes. Cells were passaged once a week and used for experiments between passages 12 and 20 from establishment [31].

### Cell Treatment with 4-Phenylbutyric Acid (4-PBA)

WT-iAstro and 3Tg-iAstro cells were treated with 4-PBA (Sant Cruz Biotechnology, Cat. sc-232961) [35, 40] for 48 h at a concentration of 3 μM, which has been chosen as a minimal concentration able to revert protein synthesis, ER-mitochondrial interaction, and secretome alterations in 3Tg-iAstro cells [33].

### Cell Lysis

After removing the medium and washing with cold phosphate buffered saline (PBS), cells were harvested in PBS and centrifuged at 1600 × *g* for 5 min. The pellet was resuspended in a lysis buffer (20 mM Tris, pH 7.4, 250 mM sucrose, 1 mM EDTA, and 5 mM β-mercaptoethanol) and passed through a 29-gauge needle at least ten times. Lysates were centrifuged at 12,000 × *g* for 15 min and the supernatants were stored at –80 °C. Protein concentration was estimated by the Bradford method [41] using bovine serum albumin as standard.

### Proteasome Activities

Proteasome activities were measured in cell lysates through fluorimetric assays, as previously reported [42], using the following synthetic substrates: Leu-Leu-Val-Tyr-AMC for ChT-L, Leu-Ser-Thr-Arg-AMC for T-L, Leu-Leu-Glu-AMC for PGPH. BrAAP activity was measured using Gly-Pro-Ala-Leu-Ala-AMC substrate in the presence of aminopeptidase-N (AP-N). The incubation mixture contained 1 μg of cell lysate, the appropriate substrate, and 50 mM Tris/HCl pH 8.0, up to a final volume of 100 μL. Incubation was performed at 37 °C, and after 60 min, the fluorescence of the hydrolyzed 7-amino-4-methyl-coumarin (AMC) was recorded (AMC, λexc = 365 nm, λem = 449 nm) on a SpectraMax Gemini XPS microplate reader (Molecular Devices, Sunnyvale, CA, USA). In order to test the 26S proteasome ChT-L, we used Suc-Leu-Leu-Val-Tyr-AMC as substrate and 50 mM Tris/HCl pH 8.0 buffer containing 10 mM MgCl_2_, 1 mM dithiothreitol, and 2 mM ATP. The effective 20S proteasome contribution to short peptide cleavage was evaluated with control experiments performed using specific proteasome inhibitors, Z-Gly-Pro-Phe-Leu-CHO and lactacystin (5 μM in the reaction mixture), and then subtracting the obtained fluorescence values from the values obtained in cell lysates.

### Cathepsins Activities

Cathepsin B and L proteolytic activities were measured in cell lysates following the protocol described by Tchoupè et al. [43] using the fluorogenic peptides Z-Arg-Arg-AMC and Z-Phe-Arg-AFC respectively, at a final concentration of 5 μM. The mixture for cathepsin B, containing 7 µg of protein lysate, was pre-incubated in 100 mM phosphate buffer pH 6.0, 1 mM EDTA, and 2 mM dithiothreitol for 5 min at 30 °C. Upon the addition of the substrate, the mixture was incubated for 15 min at 30 °C. Similarly, the mixture for cathepsin L, containing 7 µg of protein lysate, was incubated in 100 mM sodium acetate buffer pH 5.5, 1 mM EDTA, and 2 mM 37 dithiothreitol for 5 min at 30 °C and, upon the addition of the substrate, the mixture was incubated for 15 min at 30 °C. The fluorescence of the hydrolyzed 7-amino-4-methyl-coumarin (AMC, λ_exc_ = 365 nm, λ_em_ = 449 nm) and 7-amino-4-trifluoromethylcoumarin (AFC, λ_exc_ = 397 nm, λ_em_ = 500 nm) was detected on a SpectraMax Gemini XPS microplate reader. The effective cathepsin contribution to the proteolysis was evaluated through control experiments performed using the specific inhibitor CA074Me and subtracting these values from the fluorescence values obtained by analyzing cell lysates.

### Western Blotting Analysis

Cell lysates (20 μg of total proteins) were resolved by 12% or 15% SDS/PAGE and electroblotted onto PVDF membranes. Membranes with transferred proteins were incubated with the specific primary monoclonal antibody and successively with the proper peroxidase conjugated secondary antibody. Monoclonal antibodies against β5, β2, and β1 proteasomal subunits, ubiquitin, beclin-1, LAMP1, nuclear factor erythroid 2 (NF-E2)-related factor 2 transcription factor (Nrf2), histone deacetylase 6 (HDAC6), IFN-γ, and TNF-α were obtained from Santa Cruz Biotechnology, Inc. (Heidelberg, Germany). Anti-β5i, anti-β2i, and anti-β1i rabbit monoclonal antibodies were from AFFINITI Research Products Ltd, (Mamhead, UK). SQSTM1/p62 (sequestosome 1/p62) mouse monoclonal antibody was from Sigma-Aldrich S.r.L. (Milano, Italy), and the anti-LC3B antibody and cathepsin B were purchased from Cell Signaling Technology, Inc. Anti-amyloid precursor protein antibody was from Abcam (Milano, Italy). ECL western blotting detection reagents were used on a Biorad ChemiDoc MP system. Each gel was loaded with molecular weight markers in the range of 12 to 225 kDa (GE Healthcare). Glyceraldehyde-3-phosphate dehydrogenase (GAPDH) or β-actin was utilized as controls for equal protein loading: membranes were stripped and re-probed with anti-GAPDH or anti β-actin monoclonal antibodies (Santa Cruz Biotechnology, Heidelberg, Germany). Stripping buffer contained 200 mM glycine, 0.1% SDS, and 1% Tween 20. ChemiDoc-acquired immunoblot images were processed through Image J software (NIH, USA) to calculate the background mean value and its standard deviation. The background-free image was obtained subtracting the background intensity mean value from the original digital data. The integrated densitometric value associated with each band was calculated as the sum of the density values over all the pixels belonging to the selected band with a density value higher than the background standard deviation. For each band, densitometric value was normalized to the relative GAPDH or β-actin signal intensity. The ratios of band intensities were calculated within the same Western blot. All the calculations were carried out using the Matlab environment (The MathWorks Inc., Natick, MA, USA).

### Monodansylcadaverine Assay

Autophagy induction was assayed in both WT-iAstro and 3Tg-iAstro with MDC staining, using the fluorescent lysosomotropic compound that is incorporated into multilamellar bodies as a probe for labeling of autophagic vacuoles in cultured cells by fluorescence microscopy [44]. In detail, 6 × 10^5^ cells/well were seeded into 6-well culture plates. Following overnight growth, 50 μM MDC was added to cell medium. After 10-min incubation at 37 °C, cells were washed three times with phosphate buffered solution (PBS) and immediately analyzed with a Leica DMI6000B epifluorescence microscope equipped with a S Fluor 40 × /1.3 objective and a 380–420 nm filter. At least 16 areas were scanned for each sample and images are representative of three distinct subgroups for both cell lines. Fluorescence intensity was quantified using FIJI Image J software, by obtaining the corrected total cell fluorescence upon subtracting the mean fluorescence of background readings.

### ELISA Assay for Aβ Levels Determination

Aβ1–42 levels were measured in both cell lysates and cell supernatant using the mouse amyloid beta 40 and mouse amyloid beta 42 solid-phase sandwich ELISA kits (Thermo Fisher Scientific, Italia), following the kits manufacturer instruction.

### Statistical Analysis

Biochemical data is expressed as mean values ± S.D. Statistical analysis was performed using the Student *t*-test or one‐way ANOVA, followed by the Bonferroni test using Sigma-Stat 3.1 software (SPSS, Chicago, IL, USA). *p*-values *p* < 0.05 (*) and *p* < 0.01 (**) were considered to be statistically significant.

## Results

### Impaired 26S Proteasome Functionality in 3Tg-iAstro

**Fig. 1:**
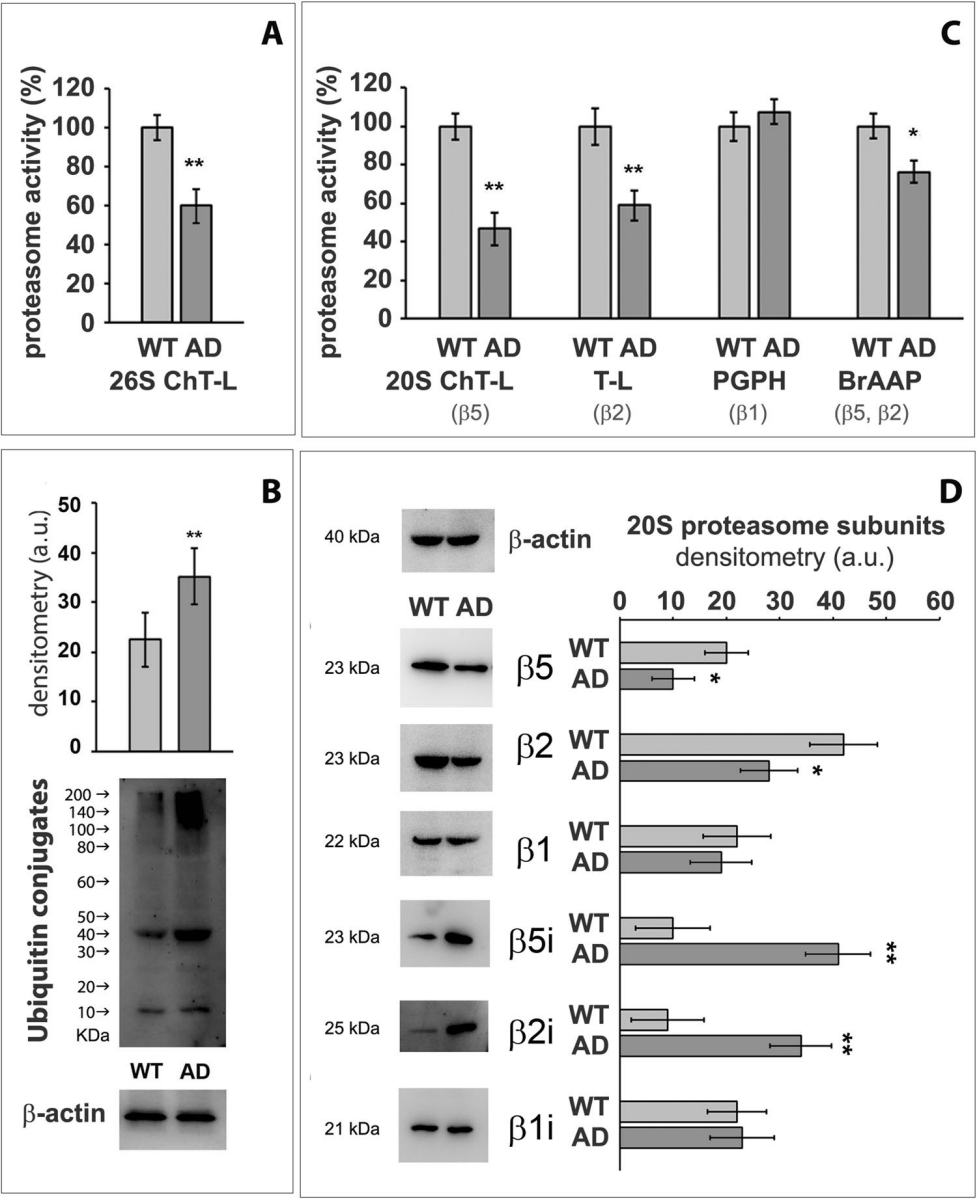
Deregulation of UPS in 3Tg-iAstro. 26S Proteasome activity in WT-iAstro (WT) and 3Tg-iAstro (AD) (panel **A**) and Western blotting of ubiquitin conjugates (panel **B**). Panel (**C**) 20S proteasomal activities measured in WT-iAstro (WT) and 3Tg-iAstro (AD) through enzymatic fluorimetry assays. From left to right chymotrypsin-like activity (ChT-L), trypsin-like activity (T-L), peptidyl-glutamyl peptide-hydrolyzing activity (PGPH), and branched-chain amino acid activity (BrAAP) of the 20S proteasome are reported. Activities are expressed as percentage of WT (data from three independent subgroups of both cell types at the same passage number). Panel (**D**) 20S proteasome subunits composition. Representative Western blots of 20S proteasome constitutive (β5, β2, β1) and inducible (β5i, β2i, β1i) subunits in WT-iAstro (WT) and 3Tg-iAstro (AD) immortalized murine astrocytes. Equal protein loading was verified by using an anti-β-actin antibody and normalized expression of the target protein is reported as arbitrary units (a.u.). The densitometry from five separate blots and an immunoblot which is representative of three distinct cellular subgroups are reported. Equal protein loading was verified by using an anti-β-actin antibody and normalized expression of the target proteins is reported as arbitrary units (a.u.). Data points marked with asterisks are statistically significant compared to the WT counterpart (at the same passage number) (*p < 0.05, **p < 0.01), upon Student’s *t*-test

A markedly reduced proteasomal 26S ChT-L activity was observed in 3Tg-iAstro compared to WT-iAstro (40% reduction, Fig. 1A). Accordingly, Western blotting results showed higher levels of ubiquitinated substrates in 3Tg-iAstro compared to WT astrocytes (Fig. 1B), in agreement with previous studies in which increased levels of ubiquitin conjugates were demonstrated to correlate with inhibited proteasomal activity [45, 46]. As UPS is able to degrade soluble misfolded proteins or small oligomers, large protein aggregates prevent degradation and inhibit UPS activity [47, 48].

### Downregulated 20S Proteasome Activities in 3Tg-iAstro

26S proteasome analysis provides an insight in the ATP-Ubiquitin-dependent protein degradation pathway, whereas 20S proteasome, lacking 19S regulatory subunits, is regulated by ubiquitin-independent mechanisms with a different target-specificity. Despite most of the research focusing on 26S proteasome, several works have demonstrated that the 20S proteasome is one of the cytoplasmic components that adapts to oxidative stress and promotes oxidized protein degradation [49, 50] preventing the formation of protein aggregates that can block both 26S proteasome and 20S proteasome independently from the ubiquitin post-translational modification.

20S proteasomal chymotrypsin-like and trypsin-like activities, attributed to β5 and β2 respectively, were the most significantly decreased in 3Tg-iAstro compared to WT-iAstro cells, in line with previous studies on a human cellular model of AD [51]. The ability to cleave after branched-chain amino acids (BrAAP activity) showed a moderate decrease in 3Tg-iAstro, whereas no significant difference was observed in PGPH activity, constitutively attributed to β1 subunit (Fig. 1C).

### Constitutive Proteasome and Immunoproteasome Were Differentially Expressed in 3Tg-iAstro Compared to WT-iAstro

Western blotting detection of constitutive and inducible proteasomal catalytic β subunits (Fig. 1D) revealed a reduced expression of constitutive 20S β subunits, specifically β2 and β5, and increased protein levels of β2i and β5i subunits in 3Tg-iAstro compared to WT-iAstro cells (*p < 0.05, Fig. 3), in agreement with data from enzymatic assays (Fig. 1C). β1 and β1i subunits were equally expressed in hippocampal astrocytes from 3xTg-AD and WT mice, in line with the stable PGPH activity in the two cellular types. Results are coherent with previous research data which demonstrated that, upon protein aggregate production during AD pathogenesis, in the attempt to cope with the increased cytoplasmic burden, cells prioritize the expression of immunoproteasome subunits to clear protein aggregates or oxidized proteins [20, 52].

UPS deregulation in 3Tg-iAstro reflects patterns already occurring in AD neural cells from both mouse and human subjects [15, 53].

### Altered Autophagic Pathway in 3Tg-iAstro

**Fig. 2:**
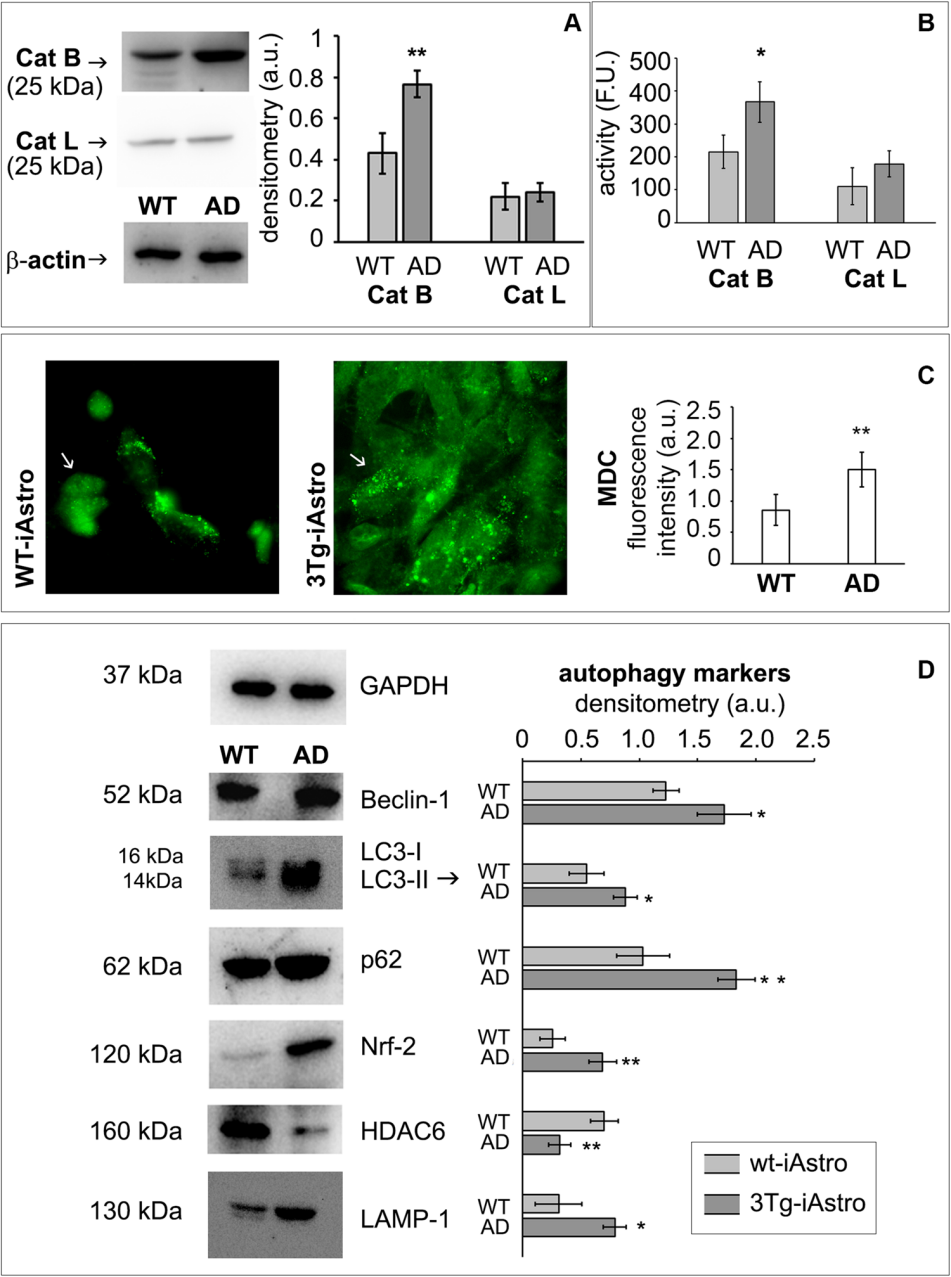
Autophagy deregulation in 3Tg-iAstro. Panel (**A**) Cathepsin B and cathepsin L representative western blotting; panel (**B**) Cathepsin B and cathepsin L enzymatic activities measured in WT-iAstro (WT) and 3Tg-iAstro (AD) and expressed as fluorescence unit (FU). Panel (**C**) Monodansilcadaverine (MDC) staining of autophagic vacuoles. 40 × microscope representative images and FIJI Image J quantitative fluorescence analysis from 16 areas for each sample and from three distinct subgroups for both cell line are reported. Panel (**D**) Autophagic markers. Representative Western blots of Beclin-1, LC3, p62, Nrf2, HDAC6, and LAMP1 in WT-iAstro (WT) and 3Tg-iAstro (AD). The densitometric analyses derive from five separate blots and three independent cell lines. For LC3, LC3-II analysis is reported [59]. Equal protein loading was verified by using an anti-β-actin or an anti-GAPDH antibody and normalized expression of the target protein is reported as arbitrary units (a.u.). Detection was executed by ECL. Data points marked with asterisks (*p < 0.05, **p < 0.01) are statistically significant compared to WT cells (at the same passage number) upon Student’s *t*-test

Upon UPS failure, the cell’s attempt to remove protein aggregates exploits autophagy, which is therefore upregulated to cope with the extended demand [54]. Accordingly, lysosomal cathepsin B expression levels (Fig. 2A) and activity (Fig. 2B) were significantly higher in 3Tg-iAstro compared to WT-iAstro, whereas no significant differences were observed in cathepsin L expression and activity (Fig. 2A-B).

MDC staining was used to deeply monitor the autophagic cascade [44], by measuring the amounts of autophagic vacuoles, the most abundant organelles contained within the dystrophic neurites that are associated to β-amyloid in senile plaques [55]. The analyses revealed an increased uptake of MDC into vacuoles in 3Tg-iAstro with the classical punctate pattern of MDC-labeled fluorescence. In detail, MDC-positive vesicles in WT-iAstro are smaller with respect to 3Tg-iAstro that contain numerous, enlarged MDC-positive vesicles (Fig. 2C).

We next evaluated the levels of the autophagy-related proteins beclin-1, LC3-II, lysosomal-associated membrane protein 1 (LAMP1), and p62. Beclin-1 plays an important role in autophagy, being involved in the enrolment of membranes to form autophagosomes [29]. LC3-II is strongly bound to the autophagosome membrane and it is considered a well-established autophagic marker [56]. LAMP1 plays an important role in lysosome biogenesis [57]. Interestingly, hippocampal 3Tg-iAstro displayed higher levels of LAMP1 and beclin-1 with respect to WT-iAstro, suggesting that autophagy was initiated (Fig. 2D). The higher levels of LC3-II in 3Tg-iAstro are in agreement with increased amount of autophagic vacuoles (Fig. 2C).

p62 is considered a scaffold protein and a signaling hub for multiple pathways including UPS and autophagy-mediated protein turnover. p62 can interact with both autophagosomes-associated LC3-II and ubiquitinated conjugates to engulf the aggregates in autophagosomes. It also interacts with HDAC6 by inhibiting its deacetylase activity to maintain acetylated α-tubulin levels and stabilize microtubules in order to enhance autophagosome trafficking. Moreover, p62 synthesis is regulated by Nrf2 [27, 56, 58].

Western blotting analyses revealed that p62 intracellular levels were markedly higher in 3Tg-iAstro, compared with WT-iAstro, suggesting an altered autophagic flux in AD, possibly due to both an increased p62 synthesis and a decreased p62 proteolysis as indicated by the enhanced Nrf2 and the decreased HDAC6 intracellular levels in 3Tg-iAstro (Fig. 2D), in agreement with studies showing that p62 synthesis can be induced by an increase in Nrf2 expression upon UPS deficiency.

These results suggest that the autophagic cascade is altered in 3Tg-iAstro, accurately reproducing AD pathology.

### 4-Phenylbutyric Acid Partially Restored Both Proteasomal and Autophagic Deficits of 3Tg-iAstro Cells

**Fig. 3:**
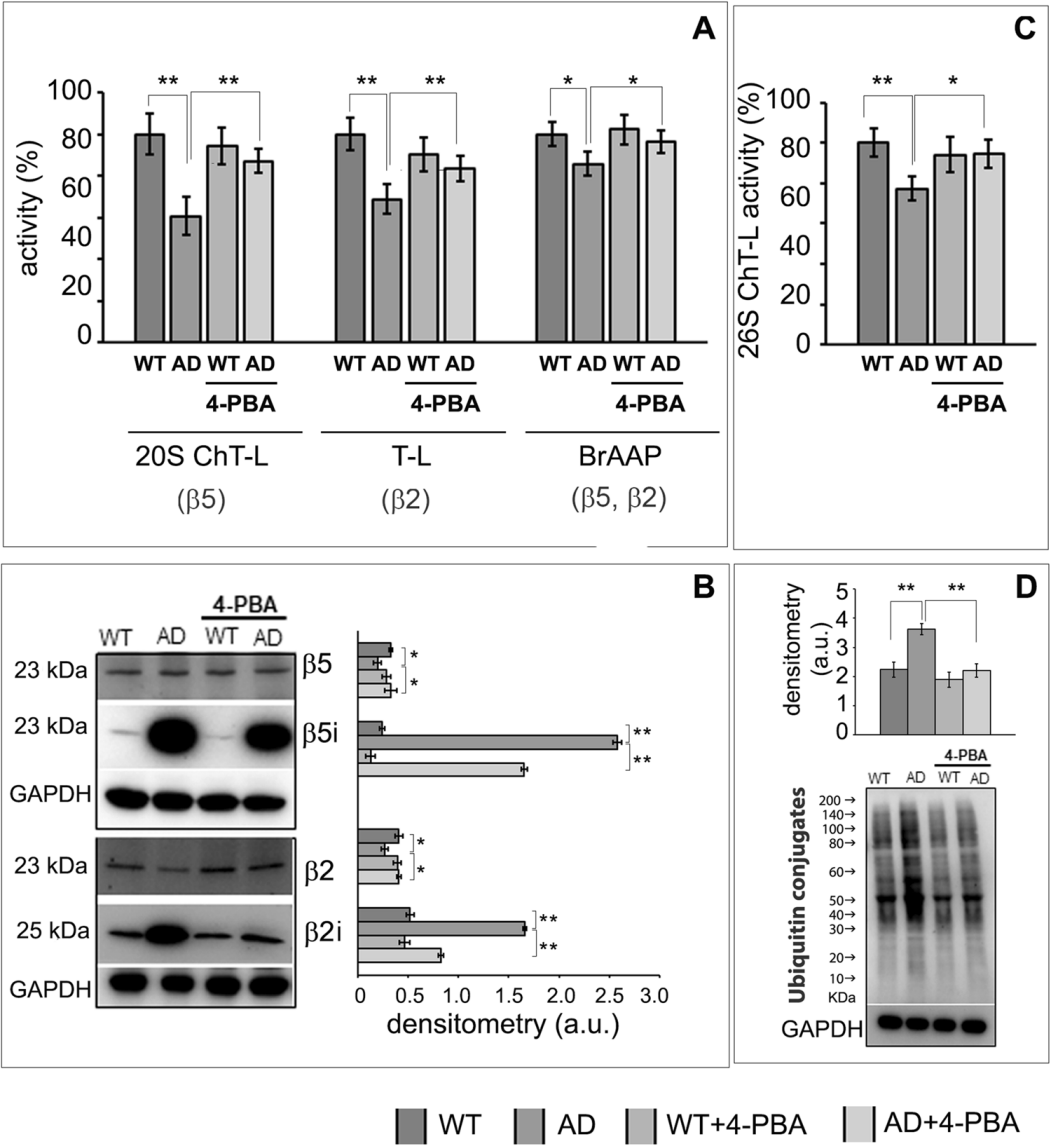
Partially restored UPS functionality in 3Tg-iAstro treated with 4-PBA. Panel (**A**) 20S Chymotrypsin-like, Trypsin-like, branched-chain amino acid cleavage (BrAAP) activities in WT-iAstro, 3Tg-iAstro, WT-iAstro treated with 4-PBA (WT + 4-PBA), and 3Tg-iAstro treated with 4-PBA (AD + 4-PBA). Values are expressed as % of control. Panel (**B**) Representative Western blots of β5, β5i, β2, and β2i in WT-iAstro, 3Tg-iAstro, WT-iAstro treated with 4-PBA (WT + 4-PBA), and 3Tg-iAstro treated with 4-PBA (AD + 4-PBA). The densitometric analyses of β5, β5i, β2, and β2i expression obtained from five separate blots and three independent experiments are shown. Equal protein loading was verified by using an anti-GAPDH antibody and normalized expression of the target protein is reported as arbitrary units (a.u.). Detection was executed by ECL. Panel (**C**) 26S proteasome activity in WT-iAstro, 3Tg-iAstro, WT-iAstro treated with 4-PBA (WT + 4-PBA), and 3Tg-iAstro treated with 4-PBA (AD + 4-PBA). Panel (**D**) Western blots of ubiquitin conjugates in treated and untreated cells. Densitometry from five separate blots and three independent experiments is reported. Anti-GAPDH was used as equal loading control. Data points marked with asterisks (*p < 0.05, **p < 0.01) are statistically significant compared to untreated cells using one‐way ANOVA, followed by the Bonferroni test

The ability of 4-PBA to restore proteolysis in 3Tg-iAstro has been investigated, because the neuroprotective effects of this FDA-approved compound are not fully elucidated [36, 60]. Upon 48-h incubation with 3 μM 4-PBA, significantly increased 20S proteasome chymotrypsin-like, trypsin-like, BrAAP activities (Fig. 3A) and 26S chymotrypsin-like activity (Fig. 3C) were observed in 3Tg-iAstro cells, with consequent reduced levels of ubiquitin conjugated proteins (Fig. 3D). 4-PBA did not significantly affect the UPS-mediated proteolysis in WT cells (Fig. 3C, D). Interestingly, upon treatment with 4-PBA, a partial restoration of the expression levels of constitutive and inducible proteasome subunits has been observed in 3Tg-iAstro (Fig. 3B).

**Fig. 4:**
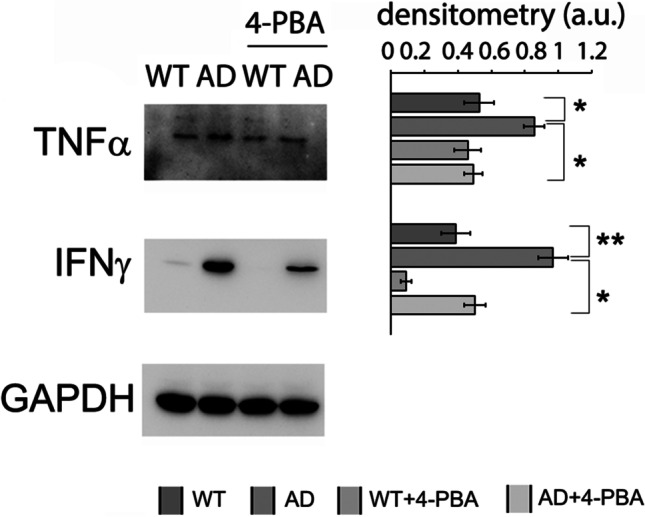
Inflammatory cytokines. Western blotting detection of TNFα and IFNγ levels in WT-Astro, 3Tg-iAstro, WT-iAstro treated with 4-PBA (WT + 4-PBA), and 3Tg-iAstro treated with 4-PBA (AD + 4-PBA). Densitometry from five separate blots and three independent experiments normalized by GAPDH is reported and expressed as arbitrary units (a.u.)

As expected, the immunoproteasome overexpression in 3Tg-iAstro was associated to increased levels of TNFα and IFNγ pro-inflammatory cytokines (Fig. 4). Interestingly, treatment with 4-PBA significantly reduced the levels of these cytokines, required for activating the innate immune response (Fig. 4), in agreement with the reduced levels of inducible proteasome subunits and with the partial recovery of proteasome functionality (Fig. 3).

**Fig. 5:**
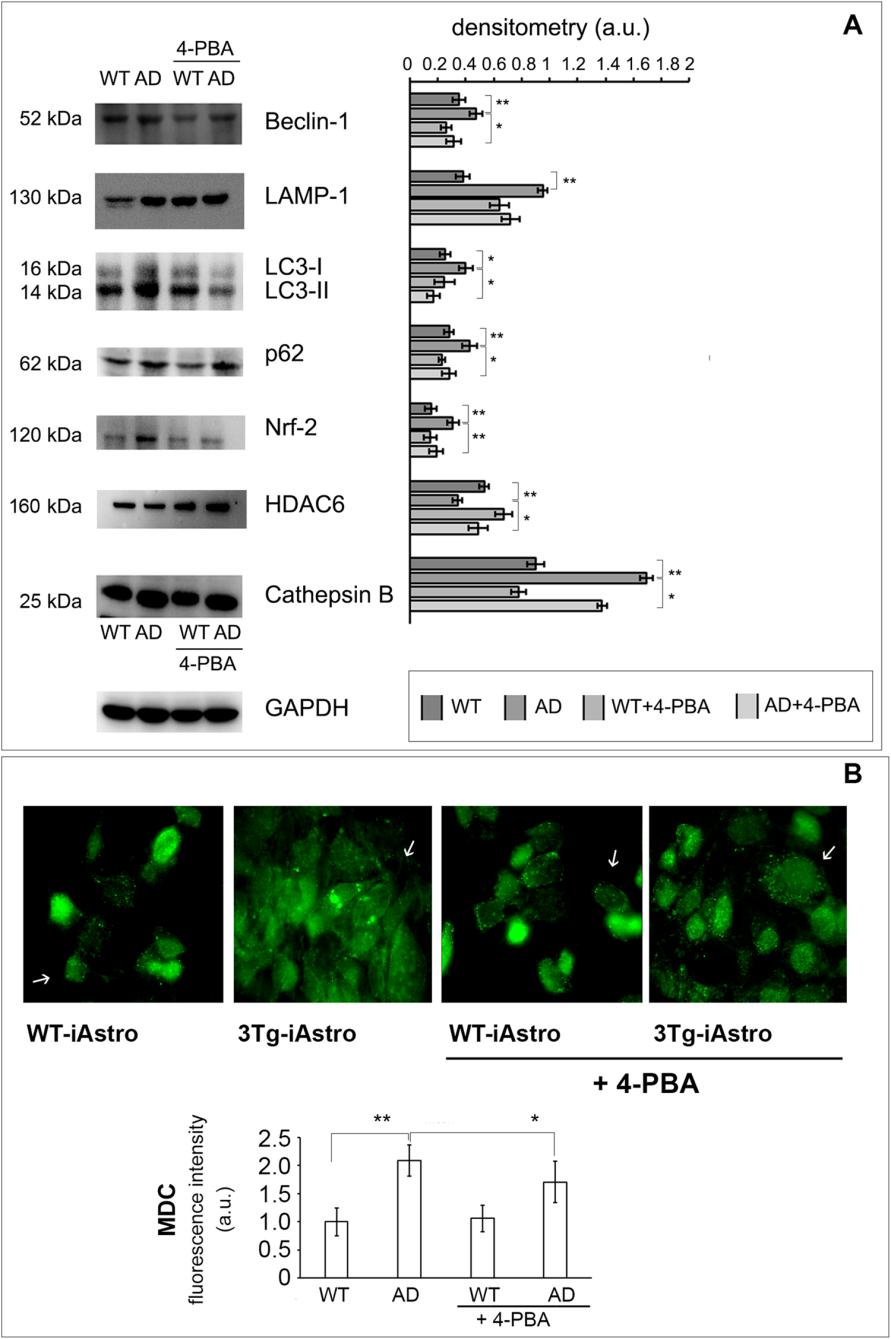
Partially restored autophagy in 4-PBA-treated 3Tg-iAstro. WT-iAstro (WT) and 3Tg-iAstro (AD) were treated or not with 4-PBA 3 μM, for 48 h respectively. Panel (**A**) Representative Western blots of Beclin-1, LAMP-1, LC3-II, p62, Nrf2, HDAC6, and Cathepsin B in WT, AD, WT treated with 4-PBA (WT + 4-PBA), and AD treated with 4-PBA (AD + 4-PBA) immortalized astrocytes. The densitometric analyses were obtained from five separate blots and three independent experiments are shown. Equal protein loading was verified by using an anti-GAPDH antibody and normalized expression of the target protein is reported as arbitrary units (a.u.). Detection was executed by ECL. Panel (**B**) MDC staining of autophagic vacuoles. 40 × microscope representative images and FIJI Image J quantitative fluorescence analysis from16 areas for each sample and from three distinct subgroups for both cell line are reported. Data points marked with asterisks (*p < 0.05, **p < 0.01) are statistically significant compared to untreated cells using one‐way ANOVA, followed by the Bonferroni test

p62, LC3-II, beclin-1, LAMP1, and cathepsin B are crucial components of the autophagy system. A significantly downregulated p62, LC3-II, beclin-1, LAMP1, and cathepsin B levels in 3Tg-iAstro cells treated with 4-PBA was observed (Fig. 5A), in agreement with previous data demonstrating that 4-PBA inhibited ER stress-induced autophagy [61]. 4-PBA significantly decreased Nrf-2 levels and increased HDAC6 levels in 3Tg-iAstro, indicating an effect on both synthesis and degradation of p62.

Interestingly, the increased MDC uptake in autophagic vacuoles in 3Tg-AD compared to WT-iAstro is mitigated by 4-PBA treatment (Fig. 5B).

Taken together, our data indicate that proteolytic pathway alterations compromise protein quality control in AD astrocytes and the chaperone may correct these alterations.

**Fig. 6:**
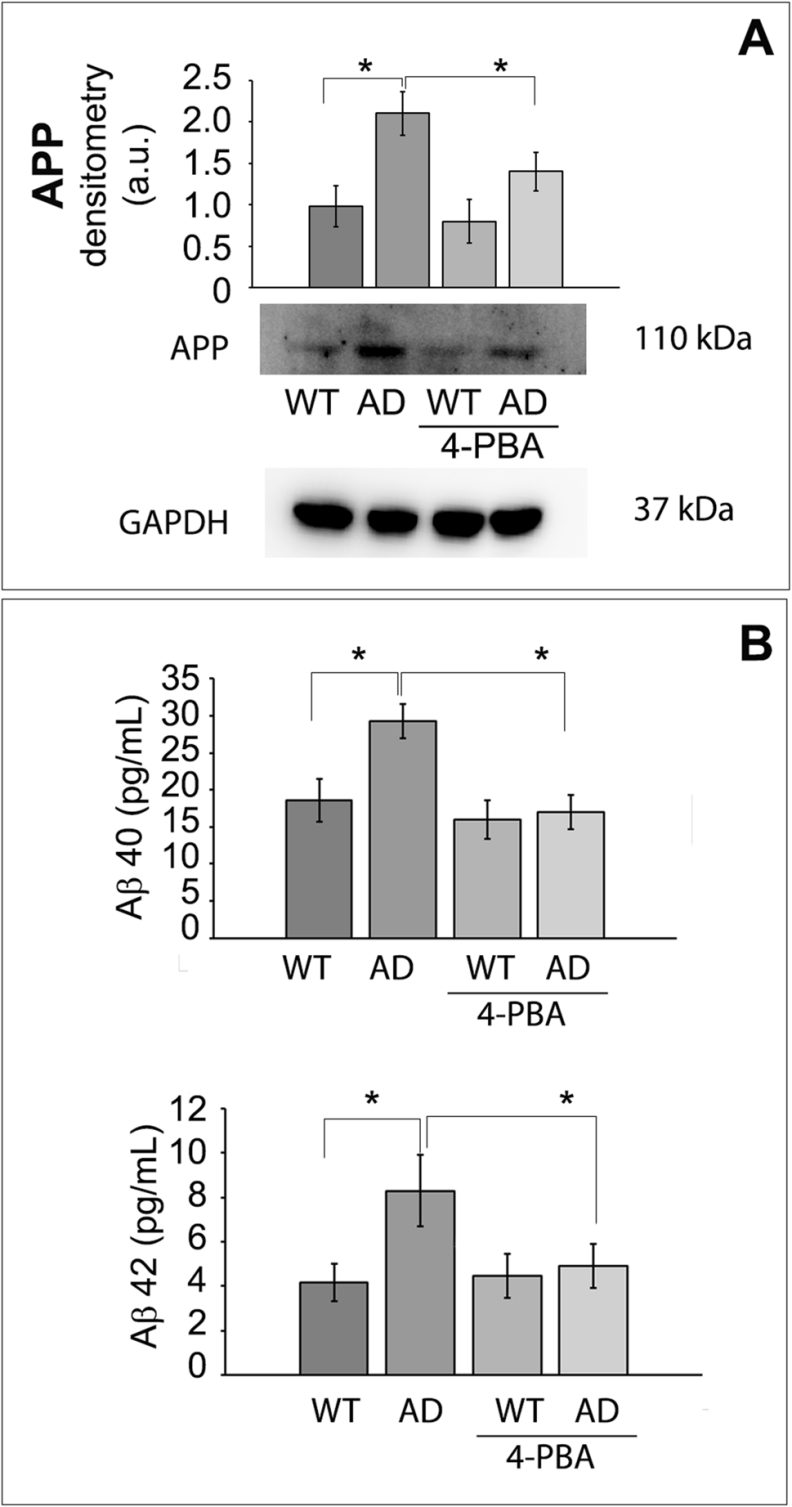
Effects on amyloid beta. Panel (**A**) Western blotting analysis of amyloid precursor protein (APP) expression in WT-iAstro and 3Tg-iAstro treated or not with 4-PBA. Densitometry from five separate blots and three independent experiments normalized by GAPDH is reported and expressed as arbitrary units (a.u.). Panel (**B**) Aβ40 and Aβ42 concentrations, expressed as pg/mL, determined by ELISA in WT-iAstro and 3Tg-iAstro treated or not with 4-PBA. Data points marked with asterisks (*p < 0.05, **p < 0.01) are statistically significant compared to untreated cells using one‐way ANOVA, followed by the Bonferroni test

Additionally, APP protein expression was higher in 3Tg-iAstro (Fig. 6A), as well as Aβ peptide concentrations (Fig. 6B) confirming that astrocytes are capable of producing Aβ, in agreement with previous reports [62] and indicating that astrocytes can contribute to total brain amyloid load. Treatment with 4-PBA significantly reduced APP, Aβ40, and Aβ42 levels in 3Tg-iAstro (Fig. 6), confirming the positive effects of the drug in the regulation of proteolysis.

## Discussion

AD can be described a proteinopathy characterized by the progressive accumulation of toxic Aβ peptides and hyperphosphorylated tau proteins in specific brain regions. Although the major focus of AD research has been neural cells, considered the main contributor of the synthesis and release of toxic Aβ species, recent evidences indicated that microglial cells and astrocytes intervene in early stages of AD pathogenic process, but more studies are necessary to provide a better understanding of their specific roles [6, 63].

In the present work, we provide the characterization of the proteolytic systems of murine immortalized hippocampal astrocytes, obtained from 3xTg-AD mice and a wild-type counterpart, to further understand the key pathways involved in protein degradation.

In spite of a number of limitations, which include their mouse origin, being generated from newborn pups, and being immortalized cells, 3Tg-iAstro cells present a series of advantages. They are a low cost and an easy-to-handle model which can be adopted to a large number of interdisciplinary and inter-laboratory investigations from single cell analysis to high throughput assays and analyses requiring large amount of starting material, such as fractionation. In particular, we used 3Tg-iAstro cells to investigate AD-related alterations of astrocytic proteostasis and found an impairment of ribosomal protein synthesis [32, 33]. In this frame, the UPS and the autophagy-lysosomal pathway are crucial for controlling cellular proteostasis [9] and we focused on both pathways to understand how their impairment contributes to the pathology. Significant differences between WT and AD immortalized hippocampal astrocytes were observed, coherently with previous research on human and murine samples [3, 15, 64] and confirming the validity of these immortalized astrocytes.

Reactive astrocytes in AD assume a heterogenous array of phenotypes which vary according to brain region and pathology. Thus, hippocampal astrocytes from a model that mimics amyloidosis in AD are likely to represent those reactive astrocytes in AD hippocampus, being the most affected brain area in AD.

Ubiquitin conjugates accumulation reflected 26S proteasomal activity impairment in 3Tg-iAstro, in accordance with previous studies demonstrating an impaired ATP synthesis in 3Tg-iAstro cells [32], since the degradation of ubiquitin conjugates by the 26S proteasome is coupled to ATP hydrolysis [65]. Moreover, since the discovery of high levels of ubiquitin in senile plaques, correlation between Aβ and 26S proteasome has been confirmed in cultured neurons and astrocytes [66, 67] and our data on APP, Aβ40, and Aβ42 levels in 3Tg-iAstro are in line with these studies (Fig. 6).

The 20S proteasome is able to degrade proteins which have not been ubiquitinated. We firstly investigated the intrinsic enzymatic activity of 20S proteasome. Despite the trend to decrease in activity, coherently with the 26S proteasome results, each activity showed a different pattern compared to WT-iAstro. ChT-L and T-L activities come from β5 and β2 subunits respectively and were both decreased in 3Tg-iAstro, while PGPH activity was not downregulated by the exposure to intracellular protein aggregates. BrAAP functionality was also slightly reduced in 3Tg-iAstro compared to the WT counterpart. This body of evidence highlights how ChT-L and T-L are preferentially impaired in AD astrocytes. This hints to a stronger involvement of β5 and β2 subunits in the attempt to remove intracellular protein aggregates, which are already known to halt the proteasome [48, 68, 69]. Interestingly, our results confirmed the shift from 20S constitutive proteasome to immunoproteasome as β5i and β2i are increased and inversely correlated with constitutive β5 and β2 expression [70]. In addition, the increase in inducible subunits can be seen as a reaction to the inflammatory state promoting the production of immunopeptides that will be exposed on MHC surface molecules. The increase in β5i and β2i correlates with mRNA levels of β and βi subunits previously measured in human hippocampal astrocytes [71] and with increased levels of IFNγ and TNFα proinflammatory cytokines in untreated 3Tg-iAstro (Fig. 4).

Autophagic flux alterations by amyloid peptides have been demonstrated in the brain of postmortem Alzheimer’s disease patients, animal models, and cell models [72]. Considering the complex and dynamic nature of the autophagy-lysosomal pathway, we monitored several markers associated with different steps of this process. Specifically, cathepsin B and cathepsin L lysosomal enzymes are differentially involved in AD development. Cathepsin B is known to possess β-secretase-like activity and thus contribute to Aβ production. Cathepsin L instead has shown the capability of increasing α-secretase activity. From literature [73, 74], when UPS is impaired, autophagy upregulation occurs to enable clearance of larger aggregates.

In this study, enhanced uptake of MDC in autophagic vacuoles was observed in AD cells (Fig. 2C). Besides, the levels of LAMP1, Beclin-1, and LC3-II as well as the level of p62 were noticeable increased in AD astrocytes compared to WT, confirming a deficient autophagic function in 3Tg-iAstro. p62 higher expression levels can be induced by an increased Nrf2 following UPS deficiency together with an inhibited autophagy reducing p62 degradation, in agreement with the accumulation of ubiquitin conjugates in 3Tg-iAstro and with previous studies [27]. Altogether, these results provide a clearer understanding of proteolytic machinery dysregulation of astroglia cells in AD pathology. Impaired proteolysis implies a reduced capacity to clear toxic, damaged, and misfolded proteins, consequently contributing to neurodegeneration. In terms of the homeostatic support to neurons and other CNS cells, impaired proteolysis may severely affect astrocytic secretome, including neurogenic and neuroprotective molecules [33, 75].

Treatment with the molecular chaperone 4-PBA partially restored proteolytic activities in 3Tg-iAstro cells. 4‐PBA interacts with hydrophobic domains of misfolded proteins preventing their aggregation [34]. Thus, this chaperone shows the beneficial role on correcting protein folding and trafficking leading to the partial recovery of UPS and autophagy functionality in AD astrocytes, with effect on the immune function, as indicated by the decreased concentrations of pro inflammatory cytokines (Fig. 4) and consequent modulation of immunoproteasome (Fig. 3B) and an effect on APP processing (Fig. 6). The higher APP protein expression in 3Tg-iAstro was significantly reduced upon 4-PBA treatment. Interestingly, significantly lower amount of both Aβ40 and Aβ42 were detected in 3Tg-iAstro upon 48-h treatment with the small chaperone confirming the positive effects of the drug in the regulation of proteolysis.

Collectively, our data shed light on proteolytic systems alterations in these reliable immortalized hippocampal astrocytes and their role in AD. Furthermore, we propose a new mechanism involved in the beneficial effect of 4-PBA in neurodegenerative disorders.

## Data Availability

The datasets generated during and/or analyzed during the current study will be available from the corresponding authors on reasonable request.
